# Neethling Strain-Based Homologous Live Attenuated LSDV Vaccines Provide Protection Against Infection with a Clade 2.5 Recombinant LSDV Strain

**DOI:** 10.3390/vaccines13010008

**Published:** 2024-12-25

**Authors:** Wannes Philips, Andy Haegeman, Nina Krešić, Laurent Mostin, Nick De Regge

**Affiliations:** 1 Sciensano (Belgium), Service of Exotic and Vector-Borne Diseases (ExoVec), Groeselenberg 99, B-1180 Ukkel, Belgium; 2 Sciensano (Belgium), Experimental Center Machelen, Kerklaan 68, B-1830 Machelen, Belgium

**Keywords:** Capripox, lumpy skin disease, recombinant strains, vaccine efficacy

## Abstract

Background: Vaccination is the main control measure to prevent Lumpy skin disease (LSD), and Neethling-based homologous vaccines have been shown to be safe and effective against infection with classical clade 1.2 strains. In 2017, recombinant clade 2 LSDV strains originating from a badly produced and insufficiently controlled vaccine were first detected in Russia. A clade 2.5 recombinant strain spread from Russia throughout Southeast Asia and caused a massive epidemic. In this study, the efficacy of three different Neethling strain-based vaccines against the recombinant clade 2.5 LSDV strain was evaluated. Methods: For each vaccine, seven bulls were vaccinated and followed for three weeks to evaluate vaccine safety. Thereafter, vaccinated animals and non-vaccinated controls were challenged with a virulent clade 2.5 strain and followed for three more weeks to evaluate vaccine efficacy. Results: Only limited adverse effects were observed after vaccination, and all vaccinated animals seroconverted and showed an LSDV-specific cellular immune response after vaccination. After the challenge, the vaccinated animals developed almost no clinical signs, and no viremia or nasal excretion was detected. This was in sharp contrast with the non-vaccinated controls, where 8 out of 13 animals developed clinical disease with clear nodules. Most of these animals also had a prolonged period of fever, a clear viremia and excreted virus. Conclusions: Neethling-based LSDV vaccines can thus be considered safe and are effective not only against clade 1.2 LSDV strains, as was proven earlier, but also against a clade 2.5 recombinant strain.

## 1. Introduction

Capripoxvirus lumpyskinpox (LSDV) forms together with Capripoxvirus sheeppox (SPV) and Capripoxvirus goatpox (GPV), the genus of Capripoxviruses within the family of Poxviridae. The genomes of these three viruses are about 150 kbp long and show high similarity among each other [1,2]. Despite their genetic similarity, they are highly host-specific. SPV and GPV mostly infect sheep and goats, respectively, although cross-infection can also occur [3,4]. LSDV, on the other hand, is only able to infect bovines, mainly cattle and water buffalo [5]. Antibodies against LSDV are nevertheless also found in different wild species, such as Greater Kudu, Impala, springbok, and giraffes, but their role in LSDV epidemiology is not entirely understood [6]. Lumpy skin disease is characterized by the formation of nodules, fever, nasal discharge, drop in milk yield and loss of weight [7,8]. Due to the associated negative socio-economic impact of disease and its transboundary nature, LSDV is listed as a World Organization for Animal Health (WOAH) notifiable disease.

Lumpy skin disease was first reported in 1929 in Zambia, from where it spread to South Africa (1944) and Kenya (1957). From 1973 onwards, it was found in western and Northeastern Africa. Between 1986 and 1988, it was found outside Africa for the first time in Kuwait [9]. One year later, it was detected in Israel, and more countries in the Middle East became affected in the following years [10]. In 2013, it was detected for the first time in Turkey and between 2015 and 2017, outbreaks occurred in Southeastern Europe, Russia and Kazakhstan [11,12,13]. Until then, only classical wild-type LSDV strains were described, consisting of two clades (1.1 and 1.2) [1]. Clade 1.1 contains isolates from the oldest outbreaks as well as LSDV Neethling vaccine strains (Live attenuated commercial LSDV vaccines), while clade 1.2 contains strains from more recent outbreaks, including outbreaks in the Middle East and Europe [13]. This phylogenetic structure changed in 2017 with the detection of the recombinant strains in Russia [14], which form a separate clade 2 [1]. Further research showed that these recombinant strains result from a recombination between LSDV Neethling and LSDV Kenyan sheep and goat pox (KSGP) vaccine strains. This probably occurred during an improperly controlled vaccine production process and batch release, and it was introduced into the field by the use of that vaccine in Kazakhstan [15]. The recombinant strains cluster in six subclades of clade 2 [1,16], and since their release in the field, clade 2.5 strains seem to have most widely spread throughout Southeast Asia.

The most differentiating feature between these new recombinant clade 2 strains and the classical clade 1 strains seems to be related to the mode of transmission. Lumpy skin disease virus is considered to be a vector-borne disease, and different vectors like stable flies and ticks have been implicated as mechanical vectors [17]. Direct and indirect transmission only seems to play a minor role for clade 1 strains, although there exists only limited data in the literature on this topic [18]. For clade 2 strains, there is a growing body of evidence that they can also spread efficiently via direct and indirect contact. This was proven in two separate in vivo studies in Russia. Five animals were infected with a recombinant strain in each of these studies. Animals were placed in the same box (direct contact) or in a different box in the same stable (indirect contact). In both cases, transmission was observed. Two different strains were used in these trials: a clade 2.1 strain for the direct transmission and a clade 2.2 strain for the indirect transmission [14,19].

The difference in transmission between the clade 1 and clade 2 strains can have an impact on control measures. Vaccination is, however, still considered the most important control measure to prevent LSD transmission and to control outbreaks [20], even for the clade 2 strains. Both heterologous and homologous vaccines have been tested and used. Heterologous vaccines make use of another Capripox virus strain (SPV or GPV) to protect against LSDV, while homologous vaccines contain an LSDV strain [21]. Although data are limited, heterologous vaccines are considered safe as they induce almost no side effects. However, field data indicated that these vaccines (at least the SPV-based) offer incomplete protection against LSDV infection [22]. Homologous vaccines, on the other hand, have proven their efficacy both in the field [23] and in controlled conditions [24]. The disadvantage associated with homologous vaccines is the side effects they sometimes induce. Most importantly, in a limited number of cases, a Neethling response has been observed, which is characterized by the formation of superficial nodules smaller than the nodules formed during an LSDV infection. These small nodules disappear after 2–3 weeks [25,26,27,28].

Although it has been clearly shown before that Neethling strain-based homologous vaccines provide good protection against clade 1 strains, the emergence of clade 2 strains with slightly altered epidemiological properties raised the question of whether they are equally effective against these new strains. Therefore, we adapted our current standardized challenge model to include a challenge with a clade 2.5 strain and evaluated the efficacy of 3 Neethling-based LSDV vaccines.

## 2. Materials and Methods

Vaccines

Three different live attenuated homologous Neethling-based vaccines were used. Two of them are commercially available and were used earlier in the field to control outbreaks and have proven their safety and efficacy against 1.1 and 1.2 LSDV strains [24]. These were, respectively, Lumpyvax (MSD, South Africa, batch 5KV24) and OBP lumpy skin disease (OBP, South Africa, batch 477). The third vaccine was the Phivax LSD vaccine (Phibro, Israel, batch M-1-3455), which is not yet commercially available. In the remainder of this manuscript, the vaccines are referred to as MSD, OBP and PHI.

Challenge virus and cell line

The recombinant challenge virus was obtained from the National Center for Veterinary Diagnosis in Hanoi, Vietnam, and belongs to clade 2.5 and is described by Mathijs et al., 2020 [29]. The challenge virus was propagated and titrated on OA3.T cells as described by Haegeman et al. (2021) [24]. Two independently produced virus stocks, both of passage 4, were used. The stock used in trial 1 had a titer of 10^6.3^ TCID_50_/mL. The virus stock used in trial 2 had a titer of 10^6.2^ TCID_50_/mL.

Animal trial set-up and ethical approval

For practical reasons, the vaccines were evaluated in two independent trials (T1 and T2) and performed in the BSL3 stables of Sciensano. The evaluation of the OBP and MSD vaccines against the clade 2.5 strain was performed in a first trial, while that of the PHI vaccine was conducted in a second trial. The set-up of both trials was highly similar.

**Figure 1:**
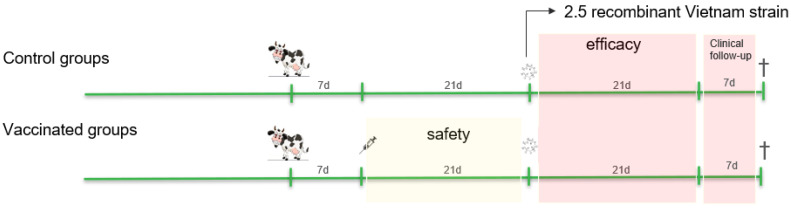
An overview of the two trials. In the first trial, the control groups consisted of 8 animals (T1) and 5 animals (T2). Each vaccinated group contained 7 animals.

Eight non-vaccinated control animals were included in the first trial, while there were 5 in the second trial. For each vaccine, seven 6-month-old Holstein Bulls (free of BVDV, IBR and BTV) were used in the study. After an acclimatization period of 7 days, vaccination with each vaccine was performed according to the manufacturer’s instructions (2 mL subcutaneously (SC) for OBP and 1 mL SC for both MSD and PHI). Twenty-one days after the vaccination, both the vaccinated and the non-vaccinated control animals were challenged with the recombinant LSDV strain. The challenge virus was inoculated intravenously (T1: 5 mL; T2: 3 mL) and intradermally. For the latter, 250 µL was injected at 4 different locations, two on each side of the neck. This resulted in an inoculation dose of 10^7.1^ TCID_50_/per animal and 10^6.8^ TCID_50_/per animal in T1 and T2, respectively. All animals (vaccinated and control animals) were sampled until 21 dpi and further clinically monitored as described below till the moment of euthanasia (27/28 dpi). An overview of the trial can be found in Figure 1.

Both animal experiments were conducted according to European Union and Belgian regulations on animal welfare in experimentation. The protocol was approved by the joint Ethical Committee of Sciensano, authorization numbers 20221024-02 and 20230627-01.

Clinical evaluation and scoring

Clinical evaluation was performed daily during the entire trial, and different parameters were scored as described in the table below. In addition, the body temperature was measured daily, and the reaction size was calculated at the site of vaccination and the site of virus inoculation (calculated as the mean diameter of the reactions at the four ID inoculation sites). An overwiew of the clinical scoring can be found in Table 1.

Sample collection

Samples were collected on day 4 in the acclimatization period, on days 0, 3, 7, 10, and 14 post-vaccination and on days 0, 3, 6, 9, 13, 16 and 21 post-challenge in the first trial and days 0, 3, 7, 10, 14, 17 and 21 post-challenge in the second trial. At each sampling, EDTA blood, serum, heparin blood and nasal swabs were collected. In case the typical nodules were observed, biopsies were taken to confirm the presence of LSDV via PCR. At the end of the trial, serum, as well as multiple organs/tissues (see Table 2), were collected to check the distribution of LSDV in the animals.

Real-time PCR analysis

The panCapX PCR panel from Haegeman et al. (2005) [30] was used to determine the presence of LSDV DNA in blood, biopsies, nasal swabs, and organs. This panel consists of three PCRs targeting three different genes (D5R, E3L, and J6R). Samples are first tested using D5R. Results around the cut-off (Ct between 37 and 42) were confirmed with the two other PCRs (E3L and J6R). Such a sample is considered positive if the Ct of one of these two additional PCRs has a Ct value of 42 or lower.

In addition, a DIVA real-time PCR (Haegeman et al., 2023) [31] was performed to investigate if the detected viral DNA originated from the vaccine (Neethling strain) or the inoculum (the clade 2.5 recombinant strain).

Serological tests

The presence of antibodies against LSDV in serum was evaluated by the commercially available ELISA from ID. Vet (ID Screen^®^ Capripox Double Antigen Multi-species) and the Immunoperoxidase Monolayer assay (IPMA) as described by Haegeman et al. (2020) [32]. No end-point titrations were conducted using the IPMA, as the serum was only tested at a 1/50 and 1/300 dilution to determine the presence of antibodies.

Interferon-gamma release assay (IGRA)

For the interferon-gamma release assay, whole blood was collected in heparin tubes. Whole blood (1.5 mL) was added to wells of 24-well plates in triplicate and stimulated with 100 µL of LSDV virus stock (10^5.2^ TCID_50_/mL), PBS (as negative control) and Pokeweed Mitogen (as positive control;160 µg/mL). Following an incubation overnight, at 37 °C and 5% CO2, the plates were centrifuged for 10 min at 500× *g* and 500 µL supernatant was collected and stored at −20 °C. Supernatants were afterward analyzed for the presence of interferon-gamma (IFN-g) using the ID Screen^®^ Ruminant IFN-g ELISA following the manufacturer’s instructions. The cut-off for the positivity of the sandwich ELISA was set to 30%. The OD values of the positive and negative controls were monitored over time to check T-cell responsiveness and identify false positive and negative results.

Statistical analysis

A Fisher-Exact test was used to verify whether there was a significant difference in the number of clinical animals in the control groups of both trials. An unpaired *t*-test with Welch’s correction was used to compare the proportion of viral DNA in the organs and tissues between the vaccinated and control animals. For both tests, *p*-values < 0.05 were considered significant.

## 3. Results

### 3.1. Unvaccinated Challenged Control Animals

Clinical signs

During the first trial, eight animals were inoculated with LSDV as unvaccinated control animals. Swollen lymph nodes and a local reaction at the site of inoculation were the first clinical signs that were observed. Starting at 6 dpi, the clinical scores started to increase due to the appearance of additional clinical signs like reduced appetite, depression, and nasal discharge. At this time point, nodules, being the most typical clinical sign of LSD, were also observed for the first time in two animals (INF02 and INF08). In the following days, additional animals developed more clinical signs and 4 more developed nodules, two at 8 dpi (INF01 and INF05), one at 9 dpi (INF06) and one at 10 dpi (INF04). The remaining two animals (INF03 and INF07) did not develop any nodules during the entire trial. These two animals also showed limited other clinical signs, reaching a clinical score of maximal 4.5. This was in contrast with the animals with nodules. Five of them reached clinical scores of at least 6, with some even reaching a clinical score of 8. There was, however, one exception, namely INF04. This animal developed only some nodules but almost no other clinical signs. The clinical signs in this animal and the animals without nodules started to disappear around 12 dpi. The remaining clinical score was caused by swelling at the inoculation site and swollen lymph nodes. A short spike in the clinical score of two of these animals was observed at 15 dpi due to an observed lack of appetite, which was probably not related to the challenge. In the other animals, the clinical signs remained present until the end of the trial.

A similar pattern was found for the temperature of the inoculated control animals. Fever was seen for the first time at 7 dpi in 4 animals. In the following two days, all of the animals had at least one day of fever. For the two animals that did not develop nodules, the fever remained limited to one or two days. In addition, the fever of two animals with nodules also remained restricted to one (INF04) or two isolated time points (INF02). The remaining animals had a prolonged period of fever of at least 7 days, reaching even up to 14 days in some. By 21 dpi, three animals still had a temperature above 40 °C.

**Figure 2:**
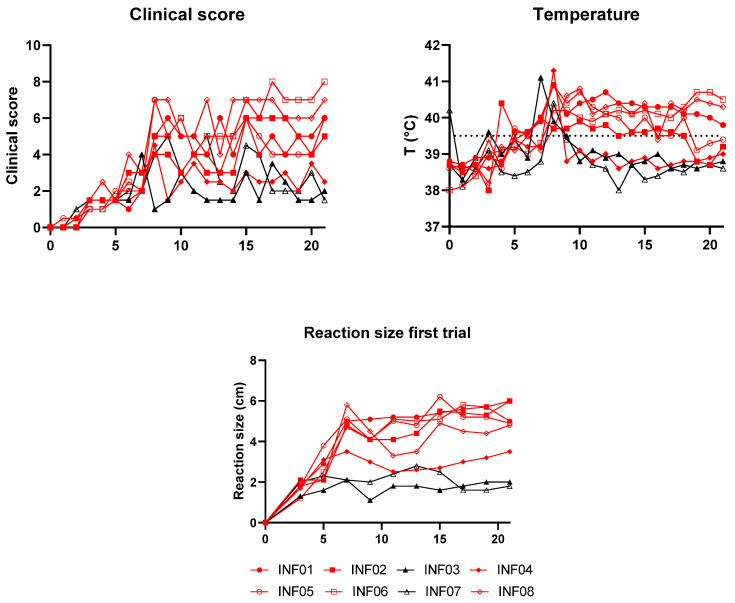
An overview of clinical data from the inoculated control animals in the first trial. The red lines represent animals with nodules; the black lines represent animals without nodules.

When looking at the local reaction at the inoculation site, there was also a clear difference between animals with and without nodules. Both animals without nodules had a local reaction that reached a maximal diameter of 2.8 cm, while this was around 5 cm in most animals with nodules. Two of these animals (INF02 and INF05) had a very strong local reaction, reaching a diameter of respectively 6.0 cm and 6.3 cm. An overview of all clinical data can be found in Figure 2.

During the second trial with 5 unvaccinated control animals, the clinical signs were similar to those observed in the first trial. A local reaction and swollen lymph nodes were the first observed clinical signs. In addition, some animals had conjunctivitis. The first nodules were again seen on day 6 after the inoculation in two animals. From that moment, the clinical score of one of these animals (INF09) increased strongly, reaching a score of 7, due to a wide variety of clinical signs, including nasal discharge, labored breathing, depression and reduced appetite. The other animal with nodules (INF13) had a less severe disease course without additional clinical signs. Although the proportion of animals that developed nodules in the second trial (2 out of 5) was smaller than in the first trial (6 out of 8), this was not significantly different (Fisher exact test; *p* = 0.29). None of the other three animals developed any nodules in the entire period post-inoculation, and clinical signs remained limited to some swollen lymph nodes, a local reaction at the inoculation site, and some diarrhea, conjunctivitis, and nasal discharge observed at isolated time points.

Concerning temperature, a first fever was seen at 5 dpi in two animals (INF09 and INF13), and nodules developed one day later. In INF13, the fever disappeared after one day, while the fever remained present for 10 days in INF09. The other animals did not develop fever in the period post-inoculation.

**Figure 3:**
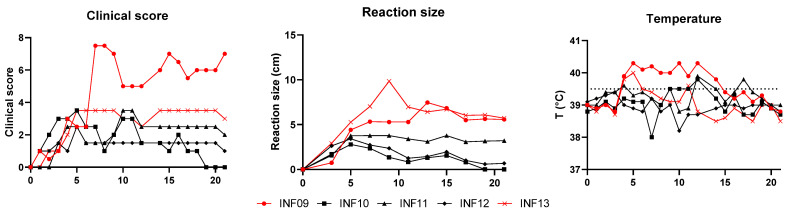
An overview of all clinical data from the control animals in the second trial. The red lines represent animals with nodules, and the black lines represent animals without nodules.

The first local reactions at the inoculation site were seen between 1 and 3 dpi. They, however, became more severe at 5 dpi, especially in INF09 and INF13, which were also the animals that developed nodules. Their local reactions reached a diameter of respectively 7.5 and 9.9 cm and were still clearly visible by 21 dpi. In the other animals, the local reaction remained limited, with a max diameter of 3.8 cm, and almost or completely disappeared by 21 dpi. An overview of all data can be found in Figure 3.

Viremia, excretion, and skin lesions

**Figure 4:**
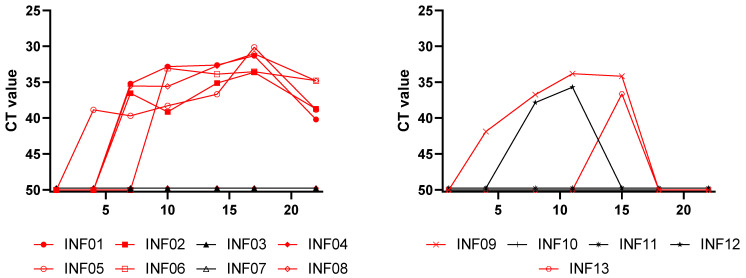
Viremia after challenge. The left panel contains the results for the first trial, while the right contains panel the results for the second trial. The red lines represent animals with nodules, and the black lines represent animals without nodules. Viremia was detected in one animal without nodules (INF12), while one animal with nodules did not have a viremia (INF04).

During the first trial, viral DNA was first detected in the blood of one animal at 3 dpi. Three days later, at 6 dpi, viremia was already detected in 4 animals, and a fifth animal became viremic at 9 dpi. The viral DNA remained detectable in the blood of these five animals until the end of the trial. No viremia was detected in the other 3 animals (INF03, INF04, and INF07), which had mild clinical disease (Figure 4).

In the second trial, LSDV was first detected at 3 dpi in one animal (INF09), which remained viremic until 14 dpi. The second animal with nodules (INF13) was only viremic at 14 dpi. A third animal (IFN12) became viremic on day 7 and stayed positive until day 10. This was one of the animals with mild clinical disease and no nodules, and it might have been a subclinical animal. The two other animals with a mild clinical disease were never found viremic.

The results for the presence of viral DNA in nasal swabs can be found in Supplementary Table S1. These data are difficult to interpret. LSDV was detected in all animals that developed generalized nodules but to different extents, with ct values ranging between 23.77 and 40.66. Viral DNA was detected for the first time in swabs from 9 dpi in one animal. At 13 dpi, 6 animals already had viral excretion. One of them without having nodules. There was no clear increasing or decreasing trend in the viral load over time. Viral DNA was also found in nasal swabs from animals without a detectable viremia and absence of generalized LSDV. Overall, these results raise the question of whether the detection of the virus in nasal swabs was an actual excreted virus or originated from contamination after contact with other animals with a more severe disease course.

In the second trial, only the nasal swabs of animal INF09—which had a prolonged fever, viremia and nodules—tested positive between 10 and 17 dpi. The positive samples were tested with the DIVA rec PCR, which confirmed that the excreted virus was the recombinant LSDV strain that was used as the inoculum. No nasal swabs from other animals tested positive.

Biopsies were collected at 9 and 13 dpi in the first trial and at 10 dpi in the second trial from the first suspected nodules and tested in PCR to confirm they were caused by LSDV. All biopsies were positive with low ct-values, indicative of a high viral load in the nodules. An overview of the ct-values of the biopsies can be found in Table 3.

Finally, LSDV distribution in the animals was investigated by collecting a selection of organs and tissues at euthanasia. These were also analyzed for the presence of LSDV DNA. The skin at the inoculation site was the only sample that tested positive in all thirteen animals, and the ct values were lower than those of other organs. The other skin samples (normal skin and skin biopsy of the scrotum) tested positive in 11 out of 13 animals. On average, 49% of the tested organs were positive for viral DNA, although clear differences were found between the animals. Among the organs tested from 2 animals (INF01 and INF08), 85% from each animal was positive for viral DNA, while others (e.g., INF05 and INF13) with a more severe disease course had only 44% and 23% positive organs, respectively. On the other hand, the absence of nodules did not necessarily mean that other organs tested negative. As an example, 62% of organs from animal INF03 were positive. 

Immune response

At the time of euthanasia in the first trial, antibodies were detected in 6 out of 8 challenged animals with ELISA (IdVET). The animals in which no antibodies were detected in the ELISA were those without nodules, INF03 and INF07. The IPMA, however, revealed the presence of antibodies in all animals, starting from 9 dpi. In the second trial, 2 animals were ELISA positive at 21 dpi, namely INF09 and INF12. Animal INF13, which had nodules as well, remained negative in the ELISA. The IPMA, however, detected seroconversion in all animals, starting between 7 dpi and 10 dpi and lasted until the end of the trial (see Supplemental Figure S1).

### 3.2. Safety of the Live Attenuated Vaccines (LAV) at Normal Dose

Clinical observations after vaccination

Fever between 4 and 6 dpv was the first clinical sign observed for all three vaccines in the period post-vaccination. Five out of 7 animals vaccinated with the MSD vaccine developed fever, while this was only the case for 2 out of 7 animals for the two other vaccines. Except for one animal that had a fever for 3 days (OBP02 from 4 to 6 dpv), the fever remained limited for a maximum of 2 days.

For each vaccine, 6 out of 7 animals developed a reaction at the site of vaccination. Four animals from the MSD and OBP vaccinated groups and 3 animals from the PHI vaccinated group had a strong reaction of at least 6 cm, with a maximum of 10.5 cm, 12 cm and 13.2 cm for the MSD, OBP, and PHI groups, respectively. The remaining animals had a local reaction that remained smaller and (almost) disappeared towards the end of the trial.

**Figure 5:**
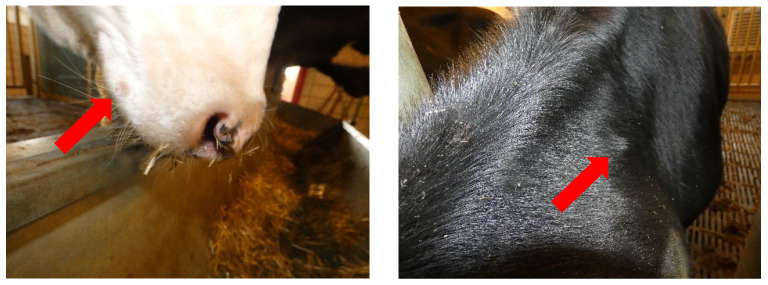
Two small nodules were observed on the nose and the shoulder of PHI03. The arrows indicate the nodules.

Other clinical signs remained limited in the period post-vaccination, with a total clinical score almost never exceeding 2.5 for any of the vaccines. This clinical score originated mostly from some swelling of the lymph nodes and the local reaction at the vaccination site mentioned above. Two animals (PHI01 and PHI03) had a clinical score of 3 and 4, respectively, at 12 dpv. For PHI01, this was due to the observation of some nasal discharge. For PHI03, this was due to swollen lymph nodes and a strong local reaction. In addition, two small nodules were observed on the shoulder and nose of this animal (Figure 5). PCR confirmed the presence of LSDV DNA in the nodule on the shoulder with a ct-value of 27.77. No biopsy was taken of the nodule on the nose because of its location. Three animals in the OBP group also developed some small nodule-like swellings in the neck region, and biopsies were taken from 2 of these 3 animals. The PCR was negative in both cases, indicating that these were not vaccine-related.

Vaccine viremia and excretion

Blood and nasal swabs were collected at regular time points post-vaccination. No viremia was detected in the entire post-vaccination period in the OBP- and MSD-vaccinated animals, while one animal from the PHI group (PHI02) was PCR positive at a one-time point (7 dpv) with a high Ct value (40.53).

The results were the opposite for the nasal swabs. None of the nasal swabs of the PHI group tested PCR positive. In contrast, viral DNA was found at different time points and in different animals in the OBP and MSD groups. The ct-values were, however, rather high and found at isolated time points.

Vaccine-induced immune response

First, an ELISA (IDVet) was performed on the serum collected at 21 dpv. Only 3 of the 21 vaccinated animals (2 in the OBP group, 1 in the MSD group) had already seroconverted by that time, according to the ELISA.

**Figure 6:**
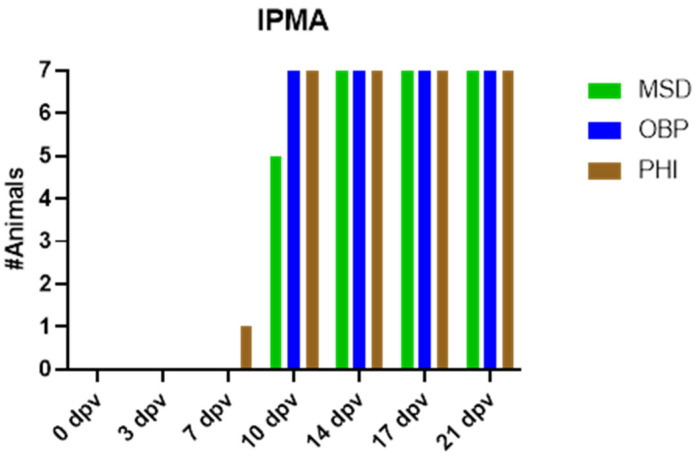
Seroconversion of vaccinated cattle according to the IPMA method.

The sera were also tested in an IPMA assay, which is known to be more sensitive than the ELISA. This method revealed that all vaccinated animals had developed antibodies against LSDV between 7 and 14 dpv, with most animals being IPMA positive by 10 dpv. In the PHI group, there was one early seroconverter at 7 dpv, while in the MSD group, two animals seroconverted by 14 dpv. An overview can be found in Figure 6.

**Figure 7:**
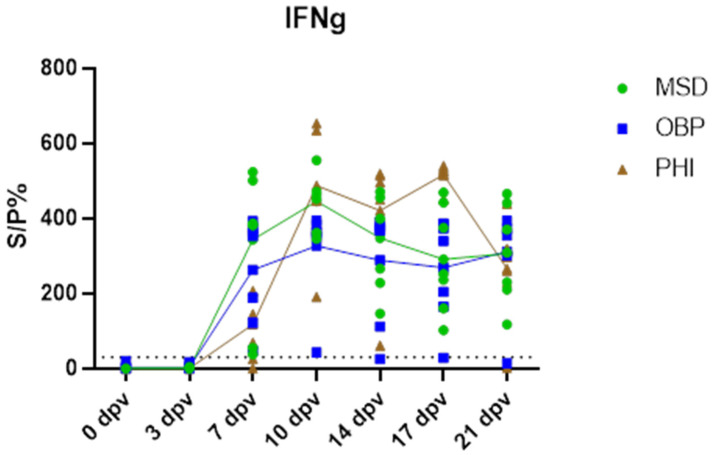
IFNg response after vaccination. Dots represent individual values, while the lines connect the mean values per vaccine.

The induced IFNg response measured by IGRA was highly similar between the three vaccines, as can be seen in Figure 7 The first positive response was seen in all 21 vaccinated animals at 7 dpv, and a plateau starting from 10 dpv remained present in almost all of these animals until the end of the vaccination period. Only two animals (one in the OBP group and one in the PHI group) were IGRA negative at 21 dpv.

### 3.3. Efficacy of the LAV Vaccines

Clinical signs in vaccinated animals upon challenge

Twenty-one days after vaccination, all animals were challenged with a clade 2.5 recombinant strain.

**Figure 8:**
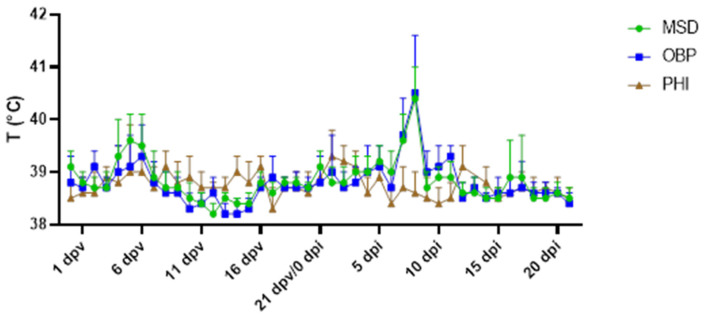
Overview of the mean temperature of the vaccinated/challenged animals. Note the small rise in temperature of around 6 dpv in all groups and the fever spike of around 7 dpi in the OBP and MSD groups.

Most animals vaccinated with the MSD (7 out of 7) and OBP (6 out of 7) vaccines developed a fever at 7–8 dpi that lasted for only one or two days. In the PHI group, none of the animals developed a fever after the viral challenge (Figure 8).

**Figure 9:**
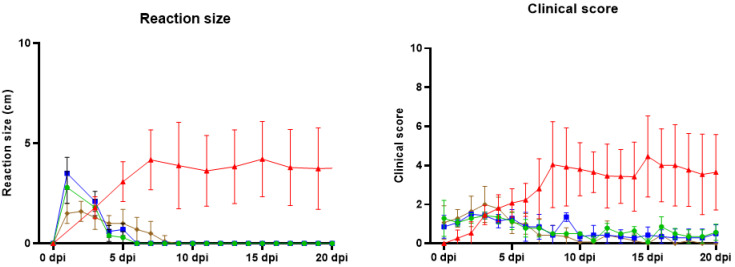
Local reaction size at the site of inoculation (left panel) and clinical score (right panel). The mean values for the MSD (Green), OBP (Blue), PHI (Brown) and Control (RED) animals are shown in the graph.

All vaccinated animals quickly developed a local reaction at the challenge site, reaching a maximal diameter of 4.3 cm, 5 cm and 2.5 cm for the MSD, OBP and PHI vaccines, respectively. This local reaction disappeared relatively fast and was smaller than 1.1 cm by 5 dpi. Besides fever and local swelling at the inoculation site, swollen lymph nodes were also regularly observed. Other clinical signs that only occurred in a limited number of animals and at isolated time points were diarrhea, conjunctivitis and a reduced appetite, meaning that these might have been unrelated to the viral challenge. No nodules were seen in any of the vaccinated animals after the challenge. An overview of the clinical score and the local reaction size can be found in the Figure 9.

Viremia, excretion and virus persistence in the vaccinated animals upon challenge

No viremia was detected in any of the vaccinated animals in the period after the challenge. The results for the nasal swabs were similar to those for the period after vaccination. None of the nasal swabs of the PHI-vaccinated animals were LSDV DNA positive. For the MSD and OBP groups, again, some nasal swabs tested positive with high ct-values and on isolated time points.

In general, the number of LSDV-positive organs/tissues was low. The mean number of PCR-positive organs in the MSD, OBP and PHI group (12%, 16% and 13%, respectively) was significantly lower (Unpaired *t*-test with Welch’s correction; *p* = 0.0005; 0.0027 and 0.0027, respectively) than in the none vaccinated control animals (48%). In general, the ct-values found in the positive organs of the vaccinated animals were also higher (lower viral load) than in the control group.

**Figure 10:**
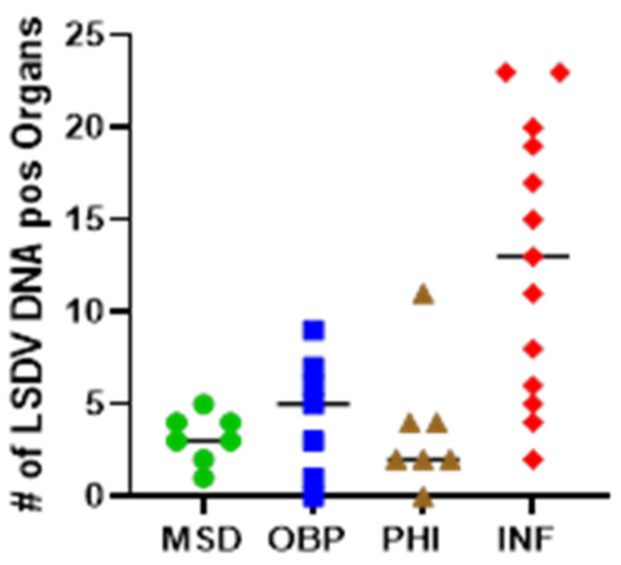
Number of qPCR-positive organs out of 27 collected organs at euthanasia per challenged vaccinated (MSD, OBP, PHI) and challenged non-vaccinated (INF) animal.

**Figure 11:**
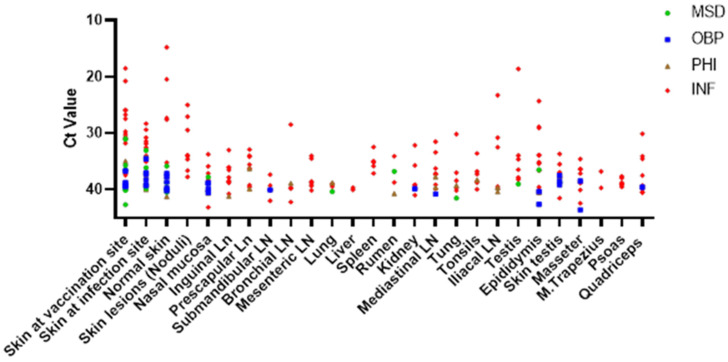
Overview of the ct-values of the positive organs at euthanasia of challenged control and challenged vaccinated cattle.

When looking at the different organs, it is clear that viral DNA was most often found in the skin samples. Sixty-six percent of the animals still had viral DNA at the vaccination site and 66% as well at the challenge site. In the normal skin, viral DNA was found in 57% of the animals. The DIVA rec was performed on some of the skin samples. The vaccine strain was only found in the vaccination site of PHI01; in all other cases, the recombinant strain was detected. An overview of the number of positive organs and the ct-values of the different organs can be found in Figure 10 and Figure 11.

## 4. Discussion

In order to perform in vivo safety/efficacy tests for LSDV vaccines against a challenge with a clade 2.5 LSDV strain, a challenge model that fulfilled the criteria recommended in the WOAH manual needed to be established. We based our study on a challenge model that was optimized before in our lab using a classical clade 1.2 LSDV wild strain [17,24,33]. When using this clade 1.2 strain, typically, about 50% of inoculated control animals developed clinical disease, although important variation between trials with that strain has been observed [24]. A similar observation was made here after the challenge with the clade 2.5 strain. In the first trial, six out of 8 (75%) animals developed nodules, and most of them progressed to a generalized LSD. In the second trial, 2 out of 5 animals developed nodules (40%). Future trials will need to point out whether this variability in the clinical outcome of the challenge model persists, but it seems in line with the reported variability in LSDV morbidity in experimental conditions [24]. Overall, the observed clinical signs were comparable between both trials, and they were in line with what was observed in previous trials with the Clade 1.2- strains [24]. The clinical disease began in both experiments with a local reaction at the challenge site and swollen lymph nodes. Around 7 dpi, a fever spike occurred, and the first nodules started to develop in some animals. The similarity in induced clinical disease and gross pathology by classical 1.2 strains and recombinant clade 2 strains was also already reported by Shumilova et al., 2023 [34] and is in line with field data [35,36,37].

Despite the similarities in clinical disease induced by strains of the different clades, a difference in the transmission patterns has recently been reported. While clade 1.1 and 1.2 strains are predominantly transmitted by vectors [17,33,38,39], efficient direct and indirect contact transmission seems to occur for clade 2 strains. We, therefore, checked the viral excretion by testing nasal swabs and hypothesized that maybe higher viral loads would be found for the clade 2.5 strain than what we found previously for the clade 1.2 strain. This was, however, not the case. The results obtained for the nasal swabs collected in the first trial raised some additional questions. At first, only swabs from animals with nodules and/or viremia were found positive. At later time points, however, swabs from animals without clinical signs or viremia also became positive with similar ct-values to swabs from clinically diseased animals. A probable explanation is that this detected DNA in the nasal swabs of the non-clinical animals originates from the environment or contact with LSDV-excreting clinical animals.

The clinical disease induced in the challenged control animals showed that the challenge model with the clade 2.5 strain worked satisfactorily. In both trials, the presence of the typical clinical signs (nodules) was observed in at least one animal, and both trials could, therefore, be considered valid safety/efficacy trials based on WOAH guidelines and be used for the evaluation of the three Neethling-based LAVs [40]. Before starting the experimental trial, all 3 vaccine batches successfully underwent an in vitro quality control whereby the identity, dose and absence of contaminants were confirmed. Next, we proceeded to the in vivo safety at normal dose testing. This had already been performed before for the MSD and OBP vaccines, while it was the first time for the PHI vaccine. During the safety evaluation, the clinical signs remained limited to a short rise in temperature around 6 dpv, swollen lymph nodes and a local reaction at the vaccination site. Besides these regularly observed mild reactions, 1 animal from the PHI vaccinated group developed a small lesion on the snout and on the shoulder. A biopsy was taken of the nodule on the shoulder, and this was PCR positive for LSDV, indicating that the nodule was indeed caused by the vaccination. It is highly unlikely that these nodules would have been detected in the field, and the reactions were not comparable to a generalized Neethling response.

Importantly, vaccination induced an immune response in all animals. Antibodies were detected in the animals at the moment of challenge, and a cellular immune response was induced based on the results of an IGRA test. This led to the expectation that the vaccination would have induced a protective immune response against challenge. This was confirmed when the animals were challenged at 3 weeks post-vaccination with a virulent clade 2.5 LSDV strain. All three vaccines provided good protection as none of the vaccinated animals developed any of the clinical signs that are normally related to an infection with LSDV, like induction of a prolonged fever or the formation of the typical nodules [41]. Furthermore, no viremia was detected in the period post-challenge and at euthanasia, the number of LSDV DNA-positive organs and the viral load present was clearly lower than in the non-vaccinated animals. The observed protection offered by the OBP and MSD vaccines was in line with what is known against the classical 1.2 strains. Overall, the results show that Neethling-based vaccines offer protection against the clade 2.5 strains that are currently circulating in Southeast Asia.

## 5. Conclusions

The challenge model that was used earlier for studies with clade 1.2 strains can also be used for studies with clade 2.5 strains. Fever and a strong local reaction at the challenge site were the first clinical signs to be observed around 6–7 dpi. This was followed by the formation of the first nodules 1 or 2 days later, and further generalization of these nodules occurred afterward. About 40–60% of the animals did not develop clinical signs, apart from a small local reaction at the challenge site, and this is in line with the variability in morbidity that was previously observed after infection with clade 1.2 strains.

The results of the safety evaluation at normal doses of the OBP and MSD vaccines confirmed earlier reports and indicated that these vaccines are safe to use. The PHI vaccine was safe as well, although two small localized nodules were observed in one vaccinated animal.

All three tested Neethling-based LAV provided protection against challenge with a recombinant clade 2.5 LSDV strain, thereby showing that these vaccines, which were used to eradicate LSDV from Europe after its incursion in 2015, could also be an important control tool to limit and potentially eradicate the disease in Southeast Asia.

## Figures and Tables

**Table 1:** Scoring system for clinical evaluations. * Neethling disease is defined as the generalized presence of small nodules over the body, which is seen about 1–3 weeks after vaccination and disappears after 1–2 weeks without leaving permanent scars.

Score	Nasal Discharges		Breathing	General Appearance	Appetite	Conjunctivitis
0	Normal		Normal	Normal	Normal	Absent
1	Mild Mucous		Increased rate	Slight depression	Reduced	Present
2	Marked mucous		Abdominal Breathing	Lethargy	No appetite	
3	Purulent		Gasping	Down		
**Score**	**Lymph nodes**		**Neethling lesions**	**Nodules**	**Swelling at the inoculation site**	
	**Prescapular**	**Inguinal**				
0	Normal		Absent	Absent	None	
1	Enlarged		Present	Present	Enlarged (0–7 cm)	
2					Very large (>7 cm)	
3						

**Table 2:** Overview of the organs and tissues collected at euthanasia.

Skin at the vaccination site	Submandibular LN	Kidney	Skin testis
Skin at the infection site	Bronchial LN	Mediastinal LN	M. Masseter
Normal skin	Mesenteric LN	Tung	M. Trapezius
Skin lesions (Noduli)	Lung	Tonsils	M. Psoas
Nasal mucosa	Liver	Iliacal LN	M. Quadriceps
Inguinal LN	Spleen	Testis	Parotid LN
Prescapular LN	Rumen	Epididymis	

**Table 3:** Overview of the Ct-values in biopsies of the first detected nodules. For INF04, no biopsies of nodules were collected.

Timepoint	INF01	INF02	INF05	INF06	INF08	INF09	INF13
9 dpi	/	18.35	17.20	/	20.58	/	/
10 dpi	/	/	/	/	/	29.86	18.54
13 dpi	13.81	22.58	17.56	16.77	25.72	/	/

## Data Availability

The main data presented in this study are available within the study itself, and other data may be made available through contact with the corresponding author.

## Supplementary Materials

The following supporting information can be downloaded at: https://www.mdpi.com/article/10.3390/vaccines13010008/s1, Figure S1: Seroconversion of the non-vaccinated animals; Table S1: Overview of the Ct-values in nasal swabs in the non-vaccinated animals.
