# Supplementary material for: Neethling Strain-Based Homologous Live Attenuated LSDV Vaccines Provide Protection Against Infection with a Clade 2.5 Recombinant LSDV Strain
Source: Vaccines (Basel). 2024 Dec 25;13(1):8. doi: 10.3390/vaccines13010008 (PMC11769152; doi:10.3390/vaccines13010008)
Supplement: Supplementary file 1 [file vaccines-13-00008-s001.zip › vaccines-3350378-supplementary.pdf]

Time-point	INF01	INF02	INF03	INF04	INF05	INF06	INF07	INF08	INF09	INF10	INF11	INF12	INF13
0 dpi	no ct	no ct	no ct	no ct	40,24	no ct	no ct	no ct	no ct	no ct	no ct	no ct	no ct
3 dpi	42,06	no ct	39,26	no ct	no ct	no ct	no ct	no ct	no ct	no ct	no ct	no ct	no ct
7 dpi	no ct	no ct	no ct	no ct	no ct	no ct	no ct	no ct	no ct	no ct	no ct	no ct	no ct
10 dpi	40,01	32,09	40,66	no ct	no ct	37,96	no ct	40,52	38,95	no ct	no ct	no ct	no ct
14 dpi	23,77	40,47	no ct	no ct	37,77	31,63	35,22	32,65	31,22	no ct	no ct	no ct	no ct
17 dpi	30,88	31,52	no ct	37,27	38,93	30,13	40,52	33,34	32,75	no ct	no ct	no ct	no ct
21 dpi	33,14	34,46	40,54	34,95	35,66	30,12	no ct	30,59	no ct	no ct	no ct	no ct	no ct

***Table S1**: Overview of the Ct-values in nasal swabs in the non-vaacinated animals after challbenge.*

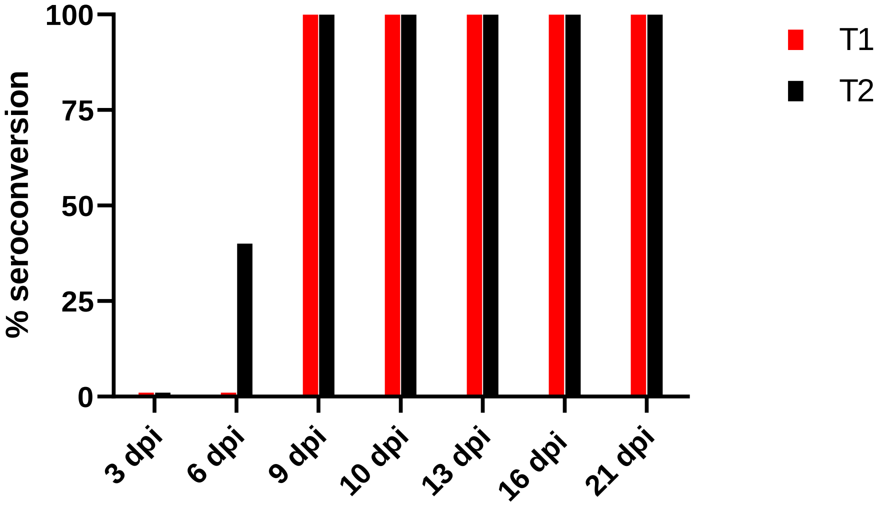

***Figure S1**: Seroconversion of the non-vaccinated animals according to the IPMA method. The %seroconversion in the the first trial (T1) is indicated in red and in belack for the second trial (T2)*
